# Global translational repression induced by iron deficiency in yeast depends on the Gcn2/eIF2α pathway

**DOI:** 10.1038/s41598-019-57132-0

**Published:** 2020-01-14

**Authors:** Antonia María Romero, Lucía Ramos-Alonso, Paula Alepuz, Sergi Puig, María Teresa Martínez-Pastor

**Affiliations:** 1 Departamento de Biotecnología, Instituto de Agroquímica y Tecnología de Alimentos (IATA), Consejo Superior de Investigaciones Científicas (CSIC), Catedrático Agustín Escardino 7, E-46980 Paterna Valencia, Spain; 2 Departamento de Bioquímica y Biología Molecular, Universitat de València, Doctor Moliner 50, E-46100 Burjassot Valencia, Spain; 3 ERI Biotecmed, Universitat de València, Doctor Moliner 50, E-46100 Burjassot Valencia, Spain

**Keywords:** Biochemistry, Molecular biology

## Abstract

Iron is an essential element for all eukaryotic organisms because it participates as a redox active cofactor in a wide range of biological processes, including protein synthesis. Translation is probably the most energy consuming process in cells. Therefore, one of the initial responses of eukaryotic cells to stress or nutrient limitation is the arrest of mRNA translation. In first instance, the budding yeast *Saccharomyces cerevisiae* responds to iron deficiency by activating iron acquisition and remodeling cellular metabolism in order to prioritize essential over non-essential iron-dependent processes. We have determined that, despite a global decrease in transcription, mRNA translation is actively maintained during a short-term exposure to iron scarcity. However, a more severe iron deficiency condition induces a global repression of translation. Our results indicate that the Gcn2-eIF2α pathway limits general translation at its initiation step during iron deficiency. This bulk translational inhibition depends on the uncharged tRNA sensing Gcn1-Gcn20 complex. The involvement of the Gcn2-eIF2α pathway in the response to iron deficiency highlights its central role in the eukaryotic response to stress or nutritional deprivation, which is conserved from yeast to mammals.

## Introduction

With the only known exception of some bacterial species^1,2^ the vast majority of living organisms depend on iron as an essential redox cofactor in fundamental cellular processes, including DNA replication and repair, mRNA translation, cellular respiration, photosynthesis and oxygen transport. Although iron is quite abundant in the Earth’s crust, its bioavailability is highly restricted under aerobic conditions because of the extremely low solubility of ferric iron at physiological pH. Thus, iron deficiency has become a widespread nutritional problem with important health and economic consequences. Therefore, the acquisition and maintenance of sufficient iron levels suppose a challenge for living organisms, which have evolved sophisticated mechanisms to modulate iron homeostasis.

The post-transcriptional regulation of gene expression plays an important role in the control of iron homeostasis in many organisms, ranging from bacteria to mammals^3–5^. In bacteria, small non-coding RNAs affect RNA stability of genes involved in iron metabolism, storage and photosynthesis^3,4^. In mammals, the binding of the Iron Regulatory Proteins IRP1 and IRP2 to Iron Responsive Elements (IRE) in the 5′- or 3′-untranslated region (UTR) of mRNAs involved in iron metabolism regulate either stability or translation^5^. The budding yeast *Saccharomyces cerevisiae* has proved to be a reliable model to characterize the mechanisms of adaptation to iron deficiency in eukaryotic cells. In *S. cerevisiae*, the Cth2-mediated decay and translational repression of specific mRNAs implicated in iron metabolism allows a metabolic remodeling that prioritizes essential over non-essential iron-dependent processes^6–8^. The mammalian Cth2 homfolog, called tristetraprolin (TTP), also post-transcriptionally modulates the expression of iron metabolism related genes, such as the transferrin receptor and genes within the mitochondrial electron transport chain, to enhance cell survival and maintain cardiac function under iron deficiency^9,10^. Most of the hitherto described post-transcriptional mechanisms that regulate iron homeostasis involve the regulation of mRNA stability, and in some cases also translation of specific mRNAs. However, little attention has been paid to the effects of iron deficiency on global translation.

Protein synthesis is possibly the most energy consuming process in the cell^11,12^. Thus, one of the primary responses of living cells to nutrient limitation or exposure to stress conditions is a general arrest of mRNA translation^13–18^. Thus, environmental cues including heat shock, oxidative and osmotic stresses, and nutritional deficiencies such as glucose or amino acid limitation, have been described to repress bulk translation^13,14,18,19^. The principal regulation of eukaryotic translation occurs at the initiation phase^20–24^. Under optimal growth conditions, eukaryotic translation initiates with the assembly of the ternary complex formed by translation initiation factor eIF2, GTP and methionyl-tRNA. At the end of the initiation step, GTP hydrolyzes into GDP and the ternary complex is disassembled. Then, the guanine nucleotide exchange factor eIF2B catalyzes the recycling of eIF2-GDP into eIF2-GTP. Upon amino acid deprivation or exposure to different stress conditions, Gcn2 protein kinase phosphorylates the alpha subunit of eIF2 (eIF2α), converting eIF2 into an eIF2B inhibitor, which causes a decrease in the levels of ternary complex and a block in global 5′ cap-dependent translation initiation. Interestingly, low ternary complex abundance enhances the translation of the *GCN4* mRNA, which encodes for a transcriptional activator of amino acid biosynthetic genes, via short upstream open reading frames (uORFs)^16^. The activation of Gcn2 kinase in response to amino acid starvation occurs through binding of uncharged tRNAs to its histidyl-tRNA synthetase (HisRS)-like domain, a process that involves the Gcn1-Gcn20 protein complex, which facilitates the transfer of the uncharged tRNA from the ribosome to Gcn2^25,26^.

Despite that the biosynthesis of multiple amino acids including leucine, lysine and methionine depends on iron, and that ribosome biogenesis and recycling requires the essential and conserved iron-containing protein Rli1, no evidence has reported yet whether protein synthesis is altered in iron-deficient yeast cells. In this work, we analyze the state of bulk translation during the progress of iron starvation in *S. cerevisiae*. Our results indicate that yeast cells activate a global repression of protein synthesis after exposure to iron starvation. This response is mediated by the Gcn2/eIF2α pathway and requires the uncharged tRNA sensing complex Gcn1-Gcn20.

## Results

### The efficiency of global mRNA translation decreases in response to iron limitation

**Figure 1:**
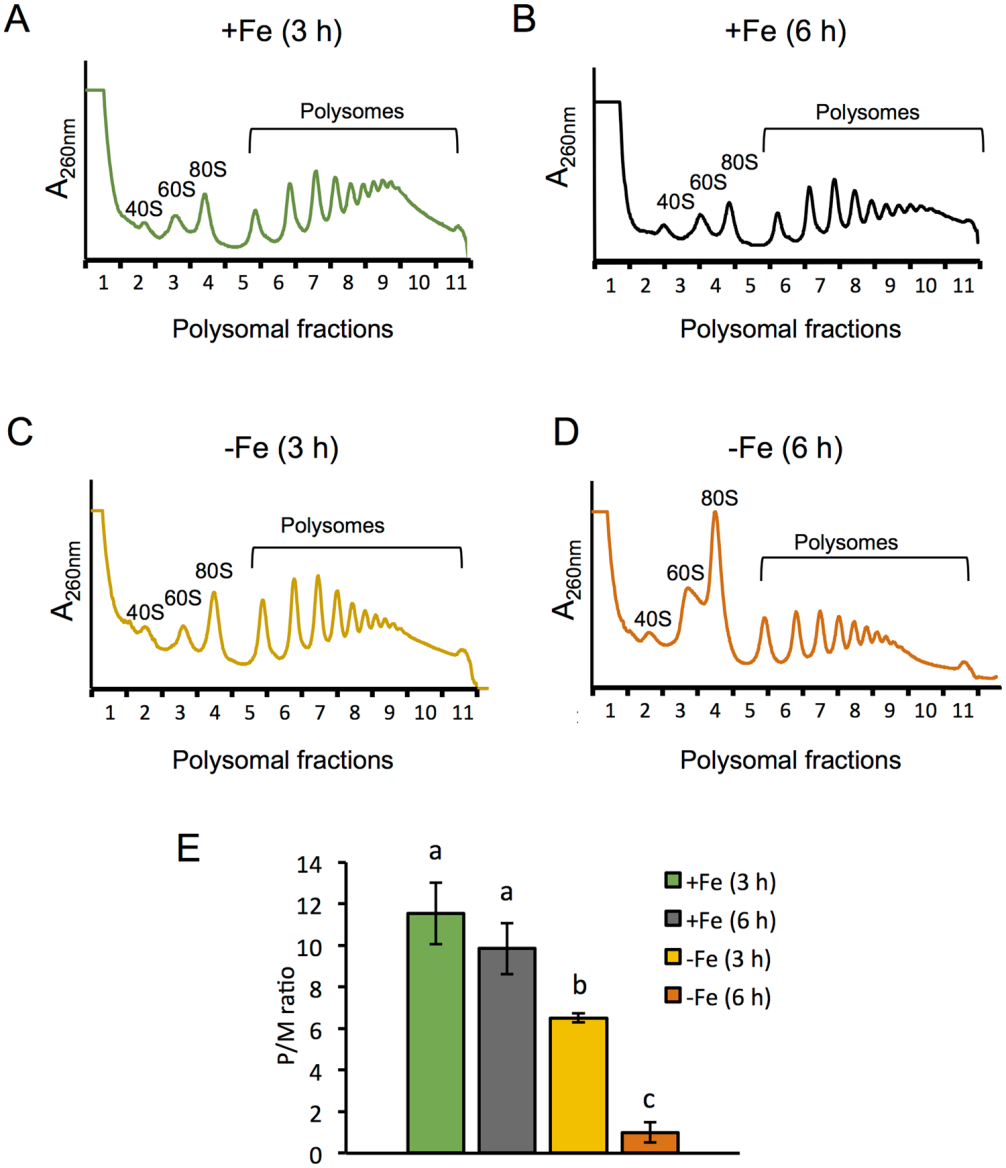
A general repression of translation occurs in response to iron deficiency. Polysome profiles were obtained from wild-type W303 strain cultivated in synthetic complete (SC) medium for at least 15 hours to reach early exponential phase (OD_600_ = 0.2), and then cells were further maintained in SC (+Fe) (**A,B**) or in SC + 100 µM BPS (−Fe) (**C,D**) for 3 (**A,C**) and 6 hours (**B,D**) as described in Methods. The A_260nm_ profiles after gradient fractionation are shown and the ribosomal subunits (40 S and 60 S), monosomes (80 S) and polysomes are indicated. Three biologically independent replicates were performed in each case, and a representative profile is shown. (**E**) The average polysomes/monosome 80 S ratio (P/M) is represented with its corresponding standard deviation. Different letters over the bars indicate statistically significant differences (*p*-value < 0.05).

To investigate whether iron deprivation alters the efficiency of bulk mRNA translation, we determined the polysome profile of wild-type W303 yeast cells cultivated in iron-sufficient (+Fe) or iron-deficient conditions (−Fe), achieved by the addition of 100 μM of the Fe^2+^-specific chelator bathophenanthroline disulfonic acid (BPS). Only a slight decrease in polysome abundance was observed after 3 hours of iron deficiency, compared to 3 hours in iron sufficiency (Fig. 1A,C,E for polysomes/80 S (P/M) ratio). More important, after 6 hours of iron depletion, we observed a significant increase of the monosomal 80 S ribosome peak and a large decrease in polysome abundance (Fig. 1D,E), compared to the polysome profile obtained in cells incubated the same hours under Fe replete conditions (Fig. 1B,E). These observations are indicative of a general repression of translation initiation during the progress of iron deficiency.

### The translational state of specific transcripts is differentially affected under iron deficiency

**Figure 2:**
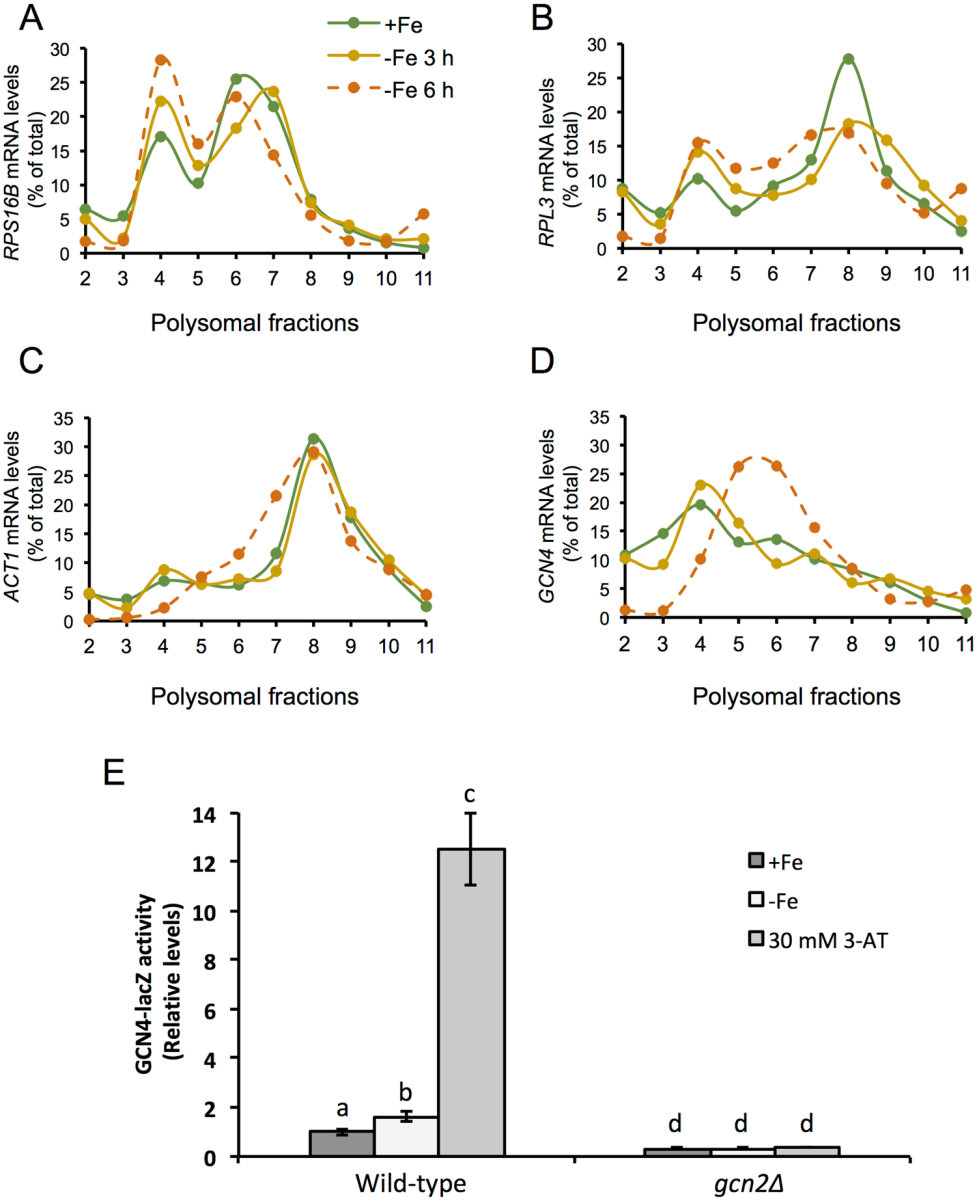
Specific mRNAs are differentially translated in response to iron deficiency. The RNA from individual fractions of the polyribosome profiles shown in Fig. 1 was extracted and the mRNA levels of *RPS16B* (**A**), *RPL3* (**B**), *ACT1* (**C**) and *GCN4* (**D**) were analyzed by RT-qPCR as described in Methods. (**E**) W303 *ura3Δ* and W303 *ura3Δgcn2Δ* cells transformed with a GCN4-lacZ reporter (p180) plasmid^27^ were cultivated in SD (+Fe), SD with 100 µM BPS (−Fe) and 3-AT, as indicated in Methods. Cells were harvested for ß-galactosidase activity assay and the values relative to wild-type *GCN2* + Fe cells represented. Mean values and standard deviations from three biologically independent experiments are shown. Different letters over the bars indicate statistically significant differences (*p*-value < 0.05).

To analyze how the global repression of translation observed under iron-deficient conditions affected specific transcripts, we studied the distribution of different mRNAs on the polysome profiles, which is indicative of their translational state. For this purpose, we extracted total RNA from the different polysomal fractions and determined the levels of some transcripts by RT-qPCR. Firstly, we analyzed the pattern of two mRNAs encoding for ribosomal proteins (RP), *RPS16B* and *RPL3*. We observed that both RP transcripts increased their association to the monosomal 80 S peak (fraction 4) along with the progression of the iron deficiency (Fig. 2A,B). Regarding the polyribosomal section of the profile, the RP transcript association either decreased (*RPL3*) and/or shifted to lighter polysomes (*RPS16B*). Both traits are characteristic of a decrease in the translation level of these mRNAs, which is in accordance to the global translational repression observed upon iron starvation. Secondly, we studied the translation pattern of the housekeeping gene actin (*ACT1*), which showed the typical profile of a highly translated mRNA under iron replete conditions (Fig. 2C). The *ACT1* polysomal pattern was mostly unaffected by the fluctuations in iron availability, and only a slight shift to lighter polysomal fractions was observed after 6 hours of iron depletion. Finally, we examined the distribution of the *GCN4* mRNA, which translation is activated when eIF2α is phosphorylated and eIF2 function is limited^16^. We observed that, under iron sufficiency, *GCN4* transcript accumulated in the monosomal fractions, a pattern that was still conserved during mild iron deficiency conditions (Fig. 2D). Remarkably, after 6 hours of iron deprivation, the *GCN4* mRNA shifted to the polysomal fractions, which evidenced ribosome enrichment and suggested an enhancement in its translation efficiency (Fig. 2D). To further address this issue, we determined β-galactosidase activity from wild-type cells expressing a GCN4-lacZ reporter construct containing the four uORFs present in the 5′-leader sequence of *GCN4* mRNA (p180 plasmid)^27^. After cultivating these cells in +Fe and −Fe conditions, we observed a slight, but significant, increase in β-galactosidase activity upon iron depletion (Fig. 2E). However, the rise in β-galactosidase activity achieved by iron depletion was not as substantial as the increase observed for 3-aminotriazol (3-AT) control treatment, which limits histidine biosynthesis leading to amino acid starvation (Fig. 2E). Taken together, these results suggest that, despite the global repression of translation that occurs when iron bioavailability is limited, the translation of specific mRNAs is differentially regulated under iron deficiency.

### The eIF2α-Gcn2 pathway is involved in the global translational arrest that occurs under iron deficiency

**Figure 3:**
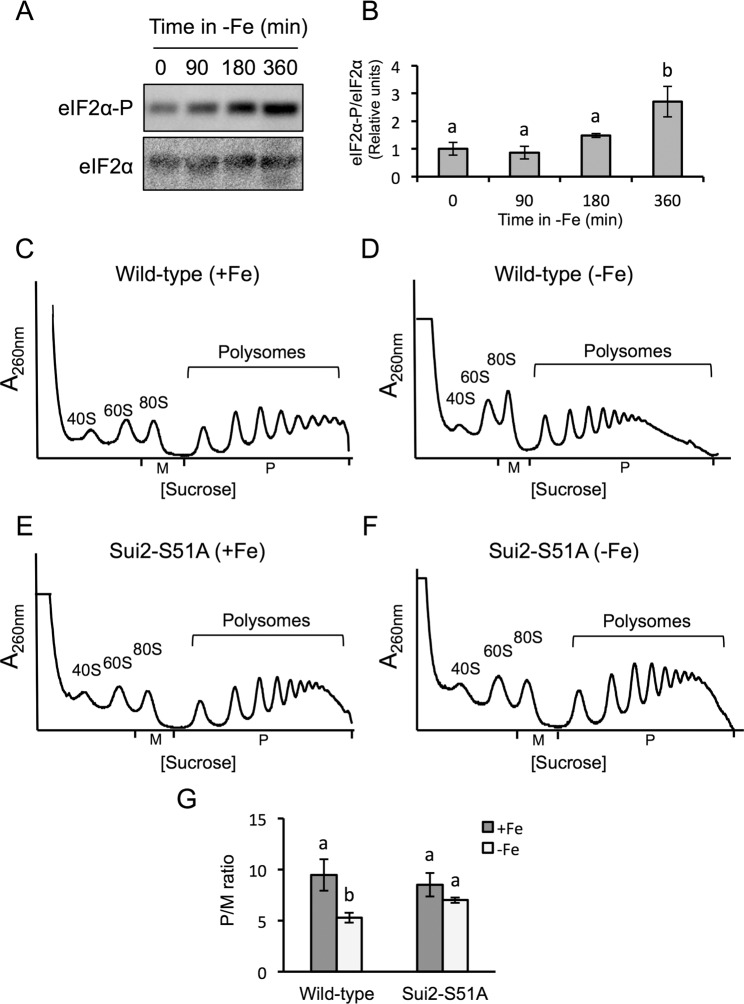
The phosphorylation of eIF2α at its serine 51 is relevant for the global translational inhibition in response to iron deficiency. (**A**) Determination of eIF2α protein levels and phosphorylation state during progression of iron deficiency (0–360 min). The levels of total eIF2α and phosphorylated eIF2α protein were determined by Western blot analyses using the anti-eIF2α and anti-eIF2α-Ser51/52 antibodies, respectively. The pictures were cropped from two independent blots where the three biological replicates were included (Supplementary Fig. S1). (**B**) Quantification of the relative levels of eIF2α-P/eIF2α is represented in panel B. The average and standard deviation of three biological replicates is shown. Different letters over the bars indicate statistically significant differences (*p*-value < 0.05). (**C**–**F**) Wild-type *SUI2* (RS-86) (**C,D**) and *SUI2-S51A* mutant (RS-88) (**E,F**) strains were cultivated in SC (+Fe) and SC with 100 µM BPS (−Fe) for 9 hours. Polysome analysis was performed as described in Fig. 1. Three biologically independent replicates were performed in each case, and a representative profile is shown. (**G**) The average P/M ratio is represented with its corresponding standard deviation. Different letters over the bars indicate statistically significant differences (*p*-value < 0.05).

Our observation that the translation of *GCN4* mRNA increases under iron deficiency suggested that the Gcn2-eIF2α pathway, which regulates translation initiation in eukaryotes, could be involved in the repression of translation under low iron. Moreover, the increase in the 80 S peak that we observe under a prolonged iron deficiency also suggested that translational repression is occurring at the initiation stage, which is the main point for eukaryotic translational control^20–24^. Under optimal growth conditions, eIF2 properly initiates translation. However, upon amino acid starvation or exposure to different stress conditions, the Gcn2 kinase is activated and phosphorylates eIF2α, which interferes with 5′cap-dependent mRNA translation^19,28–30^. Consistent with this, we observed that the expression of the GCN4-lacZ reporter construct was not up-regulated by amino acid starvation (3-AT treatment) or upon iron depletion in a *gcn2Δ* mutant (Fig. 2E). To further ascertain whether eIF2α was involved in the translational repression triggered by iron deficiency, we determined its phosphorylation state. By using an antibody that specifically recognizes the phosphorylated form of eIF2α and another antibody that binds to all eIF2α forms, we could determine that iron depletion promotes eIF2α phosphorylation without altering its total protein levels (Fig. 3A,B; Supplementary Fig. S1). These observations point to eIF2α phosphorylation as a mechanism to control protein synthesis when iron is scarce.

Previous studies have shown that Gcn2 kinase specifically phosphorylates eIF2α serine 51 to inhibit protein synthesis in response to amino acid limitation and other stresses^19,31^. Therefore, we analyzed the polysome profile of a *SUI2-S51A* mutant strain (RS-88), which expresses a non-phosphorylatable form of eIF2α^32^. Under iron-sufficient conditions, we observed that the polysomes to monosome 80 S (P/M) ratio of the wild-type (RS-86) and *SUI2-S51A* mutant strains was similar (Fig. 3C,E,G). Consistent with our hypothesis, in response to iron deficiency the P/M ratio lowered in the RS-86 wild-type strain (Fig. 3D,G), although this response was not as severe as the drop observed in the case of the W303 background (Fig. 1). Importantly, the P/M ratio observed for the *SUI2-S51A* mutant strain did not significantly decrease when iron bioavailability was limited (Fig. 3E–G). From these results we conclude that the phosphorylation of eIF2α at its serine 51 is relevant for the repression of bulk translation under iron deficiency.

**Figure 4:**
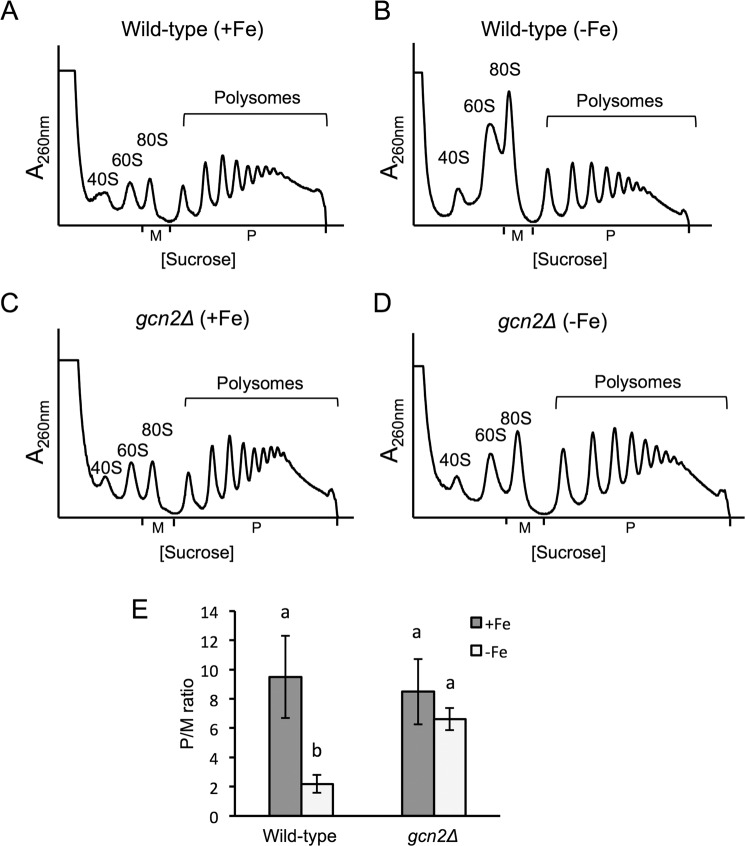
*GCN2* deletion suppresses translation inhibition during the growth in iron scarcity. Wild-type BY4741 (**A,B**) and *gcn2Δ* (**C,D**) strains were cultivated in SC (+Fe) and SC with 100 µM BPS (−Fe) for 9 hours. Polysome analysis was performed as described in Fig. 1. At least three biologically independent replicates were performed in each case, and a representative profile is shown. (**E**) The average P/M ratio is represented with its corresponding standard deviation. Different letters over the bars indicate statistically significant differences (*p*-value < 0.05).

Since Gcn2 is the kinase that phosphorylates eIF2α at serine 51, we postulated that Gcn2 could be repressing protein synthesis in response to iron deficiency. As expected, the *gcn2∆* strain displayed a growth defect in media containing 3-AT as well as a defect in eIF2α phosphorylation (Supplementary Figs. S2 and S3). Then, we determined the polysome profiles of a wild-type yeast strain and a *gcn2∆* mutant under either iron-sufficient or iron-deficient conditions. While no significant differences were observed in the polysomal profiles obtained under iron replete conditions (Fig. 4A,C,E), a significant decrease in the P/M ratio was observed under iron starvation for the wild-type strain as compared to the *gcn2∆* mutant (Fig. 4). These results evidence that the protein kinase Gcn2 plays a role in the repression of global translation caused by iron depletion. It should be noted that the role of Gcn2 in translational repression seems to be transient since, upon a longer exposure to iron deficiency, the improvement of translation of a *gcn2∆* mutant disappears and translation is inhibited to the same extent as in a wild-type strain (Supplementary Fig. S4). Taking all these results together, we conclude that the Gcn2-eIF2α pathway is involved in the repression of global translation under iron starvation.

### The repression of translation under iron deficiency depends on Gcn1

**Figure 5:**
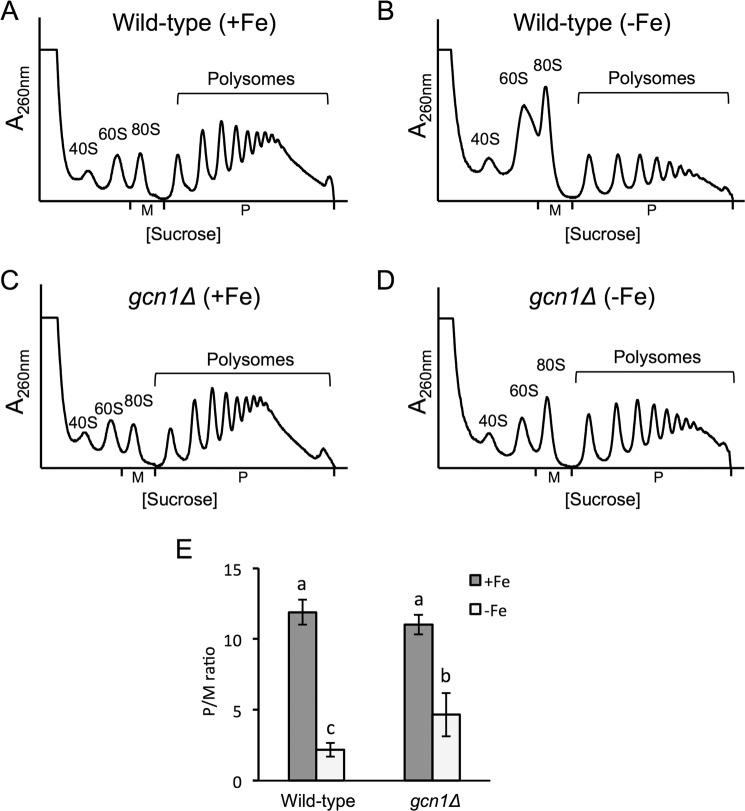
The deletion of *GCN1* improves translation during the growth in iron scarcity. Wild-type BY4741 (**A,B**) and *gcn1Δ* (**C,D**) strains were cultivated in SC (+Fe) (**A,C**) and SC with 100 µM BPS (−Fe) for 9 hours. (**B,D**) Polysome analysis was performed as described in Fig. 1. At least three biologically independent replicates were performed in each case, and a representative profile is shown. (**E**) The average P/M ratio is represented with its corresponding standard deviation. Different letters over the bars indicate statistically significant differences (*p*-value < 0.05).

The activation of Gcn2 under amino acid starvation conditions occurs through the binding of uncharged tRNAs to its histidyl-tRNA synthetase (HisRS)-like domain, a process that requires the Gcn1-Gcn20 protein complex^25,30^. To investigate whether the activation of Gcn2 kinase in response to iron deficiency was mediated by this mechanism, we analyzed the polysome profile of wild-type and *gcn1∆* cells under iron-deficient conditions. Similarly to the *gcn2∆* mutant, *gcn1∆* cells exhibited a grow defect in 3-AT-containing media and a defect in eIF2α phosphorylation (Supplementary Figs. S2 and S3). Whereas there were no differences in the P/M ratio between wild-type and *gcn1∆* mutant cells under iron sufficiency (Fig. 5A,C,E), the translation inhibition that occurred on the wild-type strain under iron deficiency (Fig. 5B,E) was partially relieved in the *gcn1∆* mutant (Fig. 5D,E). These results indicate that Gcn1 is required for the observed translation inhibition, and suggest that the mechanism that mediates Gcn2 activation under iron deficiency involves the detection of uncharged tRNAs.

## Discussion

Global repression of protein synthesis is one of the first responses of both prokaryotic and eukaryotic cells to nutritional deficiencies such as glucose or amino acid limitation, and to exposure to stress conditions like heat shock, osmotic and oxidative stress^13–15,17,18^. Opposite to glucose starvation and other nutritional stresses characterized by a rapid arrest of mRNA translation, iron deficiency is a nutritional disorder which effects are not immediate. At the initial stages of iron limitation, yeast cells activate several transcriptional and post-transcriptional regulatory mechanisms that promote the entrance of the scarce extracellular iron, the mobilization of intracellular iron stores, and the remodeling of iron metabolism in order to prioritize essential iron-dependent processes versus dispensable ones^6,8,33^. Therefore, no dramatic arrest of protein synthesis has been described after exposure to iron deficiency, which has been supported by the robust homeostasis of amino acid levels^34,35^. However, essential proteins involved in translation and ribosome recycling, such as Rli1, and multiple enzymes within various amino acid biosynthesis pathways are iron-containing proteins. Therefore, we hypothesized that upon longer iron deficiency periods, cells would not be able to maintain essential iron-dependent processes, and consequently translation would be affected. This prompted us to analyze whether a significant global arrest of translation was taking place after long exposure of yeast cells to iron limitation. For these experiments, we always maintained yeast cells at low cell densities, with an OD_600_ below or around 1.0, a condition at which cells not exposed to iron depletion maintained high translation levels. Under these conditions, we observed that mRNA translation only slightly decreased during a short-term exposure to iron scarcity (3 hours), while a more severe iron deficiency condition (6 hours) induced a dramatic repression of global translation (Fig. 1). Importantly this translational inhibition was not observed in a parallel culture grown for 6 hours under iron sufficiency with a similar OD_600_ (Fig. 1B,E), suggesting that the global translational arrest is specific of iron deficiency.

While translation is globally arrested in response to many stresses, eukaryotic cells keep or induce the translation of specific stress-responsive mRNAs, in order to adequately respond to a particular hazardous situation^36–39^. We have shown that this is also the case under iron limiting conditions, where different types of transcripts undergo distinct fates, according to their position in polyribosome profiles. While mRNAs encoding for ribosomal proteins shift towards the monosomal fractions, which is consistent with translation inhibition, the profile of the housekeeping actin gene is only slightly affected, and the uORF-containing *GCN4* transcript shifts towards the polysomal fractions (Fig. 2). Some of the mechanisms by which the recruitment of ribosomes to selective stress-responsive mRNAs happens in a general translational inhibition scenario have been elucidated, and include the presence of Internal Ribosome Entry Sites (IRES), which allow those mRNAs to skip cap-dependent translation, and the presence of uORFs in the 5′-UTR, which in the case of *GCN4* increase its translation when general translation initiation is inhibited due to low eIF2 ternary complex levels^31,40,41^. Our results support that the translation of *GCN4* could be regulated through its uORFs when iron is scarce. However, the observed shift toward the polysomal fractions does not fully correlate with a parallel increase in GCN4-lacZ expression (Fig. 2E). These results suggest that the translation of specific transcripts under iron limiting conditions depends on additional mechanisms, which require further studies.

One of the principal mechanisms that control translation during stress conditions is the phosphorylation of serine 51 in eIF2α, which turns eIF2-GDP into a competitive inhibitor of eIF2B and decreases the assembly of ternary complexes. The augmented 80 S peak and the increased translation of *GCN4* that we observed under iron deficiency were indicative of an arrest at initiation, which is the step of translation regulated by eIF2. We have demonstrated that eIF2α is increasingly phosphorylated under iron deficiency (Fig. 3A,B), and that this phosphorylation is required for the global arrest of translation that occurs in response to iron scarcity (Fig. 3C–G). Gcn2 is the sole kinase that phosphorylates the alpha subunit of eIF2 in *S. cerevisiae*. Consistent with this, a *gcn2∆* mutant did not show the eIF2α phosphorylation and the global inhibition of translation displayed by the wild-type strain upon iron deficiency (Supplementary Fig. S3 and Fig. 4). Therefore, we conclude that phosphorylation of eIF2α by Gcn2 is necessary for this translational arrest. However, when the iron deficiency persists for a longer period, other mechanisms contribute to translational inhibition since no differences in the polysome profile are observed between *gcn2∆* and wild-type cells after 12 hours of iron depletion (Supplementary Fig. S4).

The role played by Gcn2-dependent phosphorylation of eIF2α in the global inhibition of translation that yeast cells undergo in iron deficiency seems to be conserved. In mammals, iron deficiency limits heme synthesis, which activates heme-regulated eIF2α kinase (HRI), one of the four mammalian eIF2α kinases that phosphorylate eIF2α. This causes a decrease in bulk translation, down-regulating the synthesis of globins while increasing the translation of specific transcripts, such as HbF (coding for fetal hemoglobin) in certain anemias^42–45^. The targeted deletion of HRI causes an improvement of global translation, similar to that observed for the deletion of yeast *GCN2*, which increases globin translation^42^. In addition to HRI, mammalian cells possess three other eIF2α kinases, which are expressed in different tissues and activated by specific physiological stresses, namely GCN2, activated by nutrient deprivation; PKR, which responds to viral infection; and PERK, activated by endoplasmic reticulum (ER) stress^28,46–50^. The phosphorylation of eIF2α by any of these stress-activated kinases induces a stress-resistant state that results from the attenuation of global translation, diverting amino acids to other metabolic necessities, while selectively enhancing translation of ATF4, the mammalian *GCN4* counterpart. This stress response pathway, of which eIF2α is the central node, has been termed the Integrated Stress Response (ISR)^51,52^, and was initially outlined in yeast as the General Amino Acid Control (GAAC), a response to amino acid depletion that induced the expression of amino acid biosynthetic genes^53^. Since then, Gcn2 has been described to be activated by a wide variety of stress conditions in yeast, including purine starvation, glucose limitation, ER stress, ethanol, heat shock, salt stress, acid exposure, and oxidative stress^17,54–58^. The present study adds iron starvation to the list of conditions that activate Gcn2. Thus, the ISR could be considered a stress response conserved from yeast to mammals.

The activation of Gcn2 by amino acid starvation requires both the interaction with the Gcn1-Gcn20 complex and its binding to uncharged tRNAs through its histidyl-tRNA synthetase (HisRS)-like domain^25,30^. The current model proposes that Gcn1 binds to the ribosome, close to the A site, mediating the transfer of the uncharged tRNA to Gcn2^26^. In yeast, the activation of Gcn2 under other stresses, such as oxidative stress, depends on Gcn1 and Gcn20 regulatory factors, and therefore on the sensing of tRNA levels^17^. Gcn20 is not essential for Gcn2-mediated *GCN4* translational control under glucose limitation, but it is required for full repression^55^. By performing polysomal profiles of *gcn1∆* mutant cells, we show that Gcn1 is important for the repression of protein synthesis under iron deficiency (Fig. 5). Therefore, we conclude that the sensing of uncharged tRNAs is the main mechanism that activates Gcn2 in response to iron deficiency. This seems to be the main way of activation of Gcn2 in *S. cerevisiae*, fission yeast, and mammals, in response to a wide range of stresses, even those where no obvious accumulation of uncharged tRNAs occurs, such as UV treatment. This common mechanism of Gcn2 activation by different types of stress underscores the relevance of the ISR as a general and conserved way of eukaryotic cells to cope with stress and nutrient limitation^52^. In spite of many common features, the concrete ISR-mediated mechanisms that determine cell fate in response to each stress are only starting to be deciphered in mammalian cells, and therefore, understanding which specific mechanisms are activated downstream of Gcn2-eIF2α-Gcn4 in iron scarcity remains an intriguing challenge that needs to be addressed.

## Methods

### Yeast strains and growth conditions

The *S. cerevisiae* strains used in this study are listed in the Supplementary Table S1. Yeast cells were cultivated in synthetic media to exponential phase as indicated. To induce iron deficiency, 100 μM of the Fe^2+^-specific chelator bathophenanthroline disulfonic acid (BPS; Sigma), whereas 3-aminotriazol (3-AT) treatment was used to induce amino acid starvation. Aliquots were isolated at the indicated times for further analyses.

### Polyribosome profiles analyses

Yeast cells were cultivated to exponential phase in SC or SC + 100 μM BPS, and aliquots were taken at the indicated times. Polyribosome profiles analyses were performed as previously described^8,38^. For the RNA analyses of the polysomal fractions, *lys* and *spo* mRNAs from *Bacillus subtilis* were added to 200 μL of each fraction before extraction at a concentration of 3 ng/μL. RNA was extracted with the SpeedTools Total RNA Extraction kit (Biotools B&M Labs) and a DNAse treatment was performed after the RNA elution step. Specific primer pairs were used to analyze mRNAs by RT-qPCR (Supplementary Table S2), and values were represented as a percentage of the total. All the values were normalized with the spiked-in mRNA levels of *B. subtilis lys* and *spo*. At least three biological replicates were performed for each polyribosome profile, and a representative profile is shown. The average polysomes/monosome 80 S (P/M) ratio was represented with its corresponding standard deviation.

### *GCN4-lacZ* expression assays

Cells transformed with the GCN4-lacZ reporter plasmid (p180)^27^ were cultivated overnight in synthetic minimal medium (SD), and then diluted and reinoculated in SD (+Fe) or SD + 100 µM BPS (−Fe) for 6 hours, and SD + 30 mM 3-AT for 5 hours. Cells were harvested and ß-galactosidase activity was measured in permeabilized cells as previously described^59^.

### Protein analyses

Yeast proteins were extracted and analyzed by Western blot as previously reported^8^. The primary antibodies used in this study included anti-eIF2α (from John M. Zaborske) and anti-eIF2α-Ser51/Ser52 (Cell Signaling Technology). Images were obtained with an ImageQuant LAS 4000 mini biomolecular imager, and specific signals were quantified with ImageQuant TL analysis software (GE Healthcare Life Sciences).

### Statistical analyses

The data and error bars represent the average and standard deviations of at least three independent biological samples. Two tailed t-student tests were applied to evaluate statistical significance. When the statistical test indicated that two means are significantly different from each other (*p*-value < 0.05), the bars were labeled with different letters.

## Supplementary Material

Supplementary Material.
