# Supplementary material for: Global translational repression induced by iron deficiency in yeast depends on the Gcn2/eIF2α pathway
Source: Sci Rep. 2020 Jan 14;10:233. doi: 10.1038/s41598-019-57132-0 (PMC6959253; doi:10.1038/s41598-019-57132-0)
Supplement: Supplementary file 1 — Supplementary Material. [file 41598_2019_57132_MOESM1_ESM.pdf]

Supplementary Information for the manuscript "Global translational repression induced by iron deficiency in yeast depends on the Gcn2/eIF2α pathway"

Antonia María Romero[1], Lucía Ramos-Alonso[1], Paula Alepuz[2,3], Sergi Puig[1]* and María Teresa Martínez-Pastor[2]**

[1]Departamento de Biotecnología, Instituto de Agroquímica y Tecnología de Alimentos (IATA), Consejo Superior de Investigaciones Científicas (CSIC), Catedrático Agustín Escardino 7, E-46980, Paterna, Valencia, Spain.

[2]Departamento de Bioquímica y Biología Molecular, Universitat de València, Doctor Moliner 50, E-46100, Burjassot, Valencia, Spain.

[3]ERI Biotecmed, Universitat de València, Doctor Moliner 50, E-46100, Burjassot, Valencia, Spain.

*spuig@iata.csic.es

**maria.teresa.martinez@uv.es

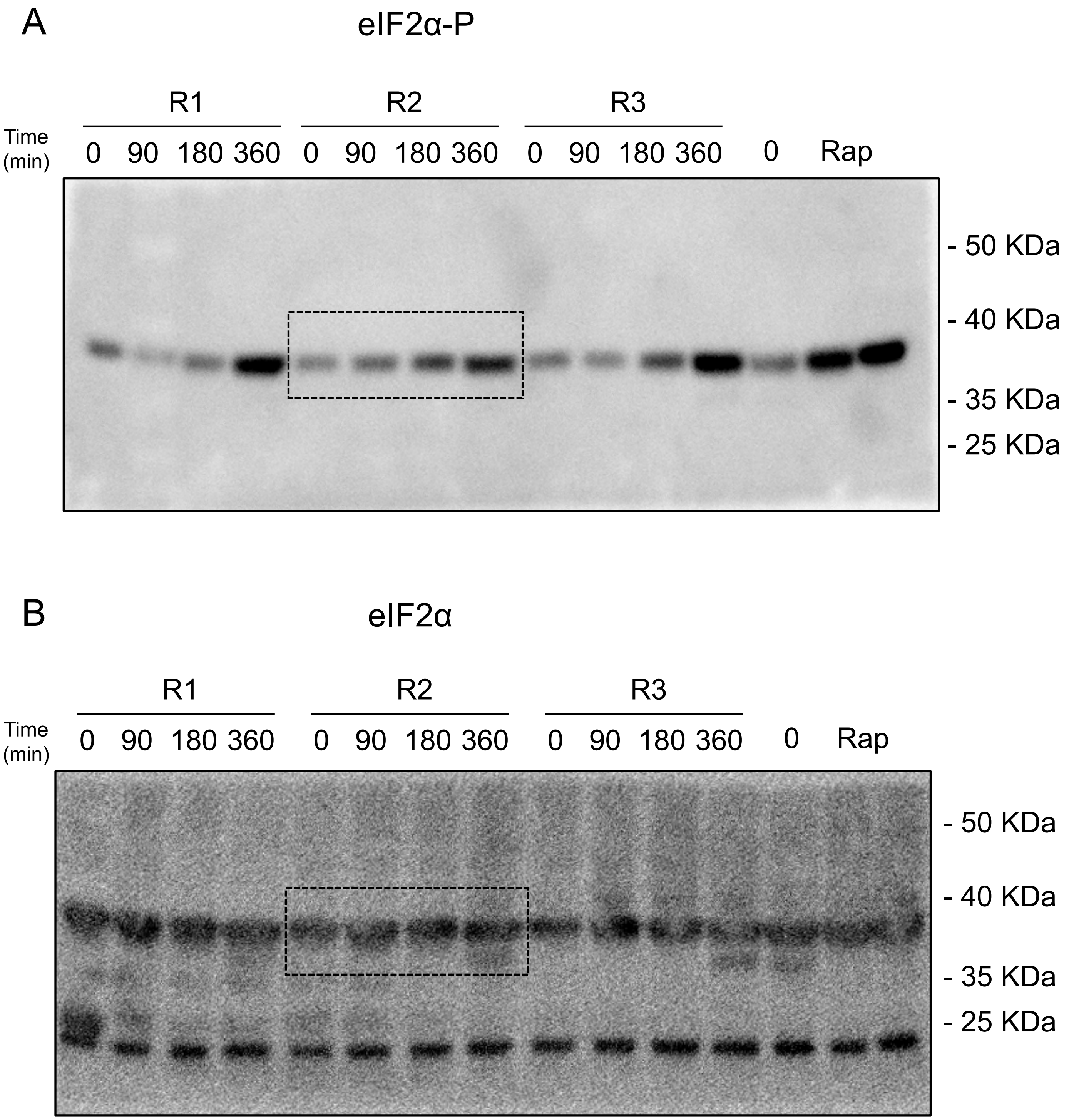

Supplementary Figure S1. Full blot image of Figure 3A with cropped regions marked with rectangles**.** The blots show the phosphorylated eIF2α (A) and total eIF2α protein of three biological replicates from cells grown under iron deficiency for the indicated times (R1, R2 and R3). The three lanes on the right show the phosphorylated eIF2α (A) and total eIF2α protein (B) of cells untreated (0) or treated with 1 μg/mL Rapamycin for 15 min (Rap), as a positive control of eIF2α phosphorylation.

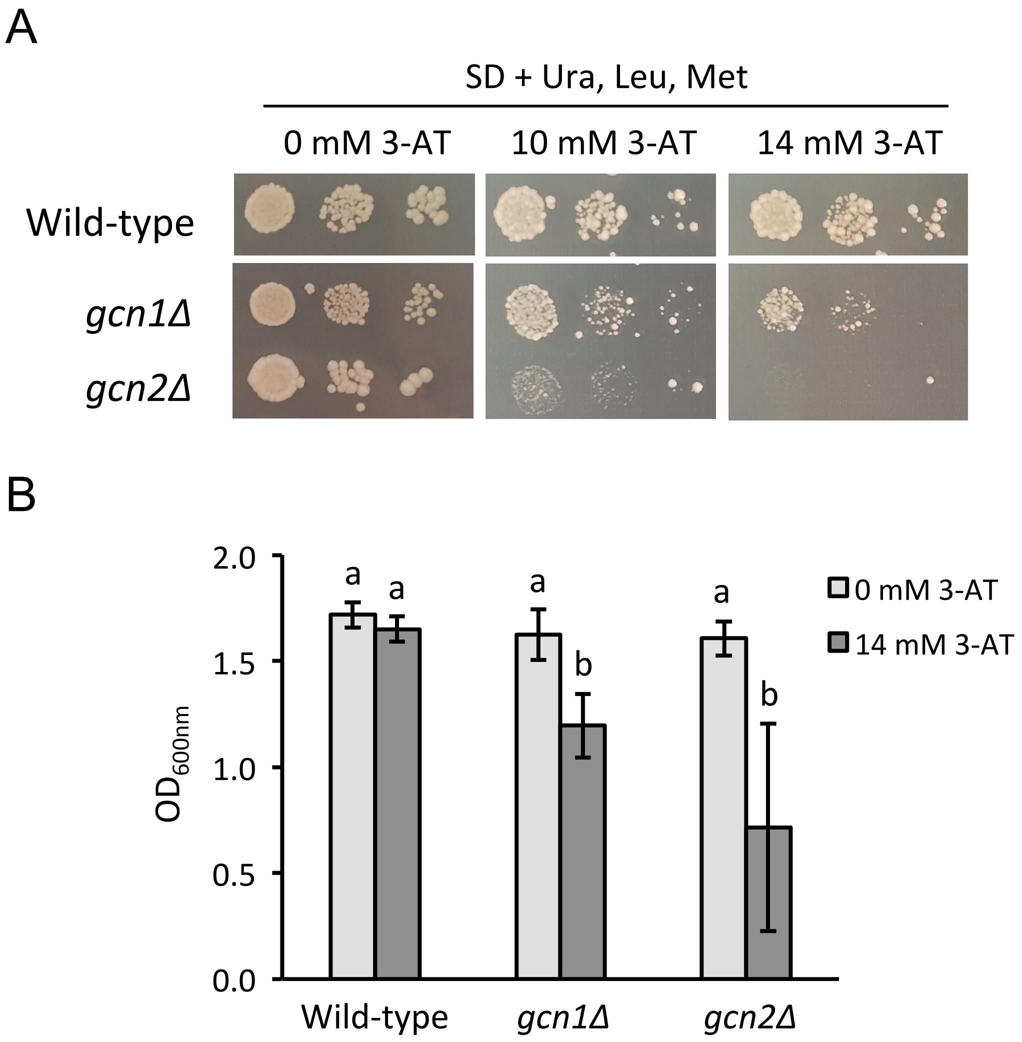

Supplementary Figure S2. The *gcn2*Δ and *gcn1*Δ mutant strains display a growth defect in the presence of 3-aminotriazole. Wild-type BY4741, *gcn2*Δ and *gcn1*Δ cells were transformed with pRS413 plasmid, which contains the *HIS3* gene. **(A)** Cells were cultivated overnight in liquid SC-His medium and then spotted in 1:10 dilutions, starting at OD at 600 nm of 0.1, on solid SD + Ura + Leu + Met plates that contained increasing 3-aminotriazole (3-AT) concentrations. Plates were incubated for 3 days at 30ºC and photographed. **(B)** Cells were cultivated in liquid SD + Ura + Leu + Met medium without or with 3-AT in a Spectrostar Nano absorbance reader (BMGLabtech) for 3 days at 28ºC, and the final OD at 600 nm was registered. Mean values and standard deviations from three biologically independent experiments are shown. Different letters over the bars indicate statistically significant differences (p-value < 0.05).

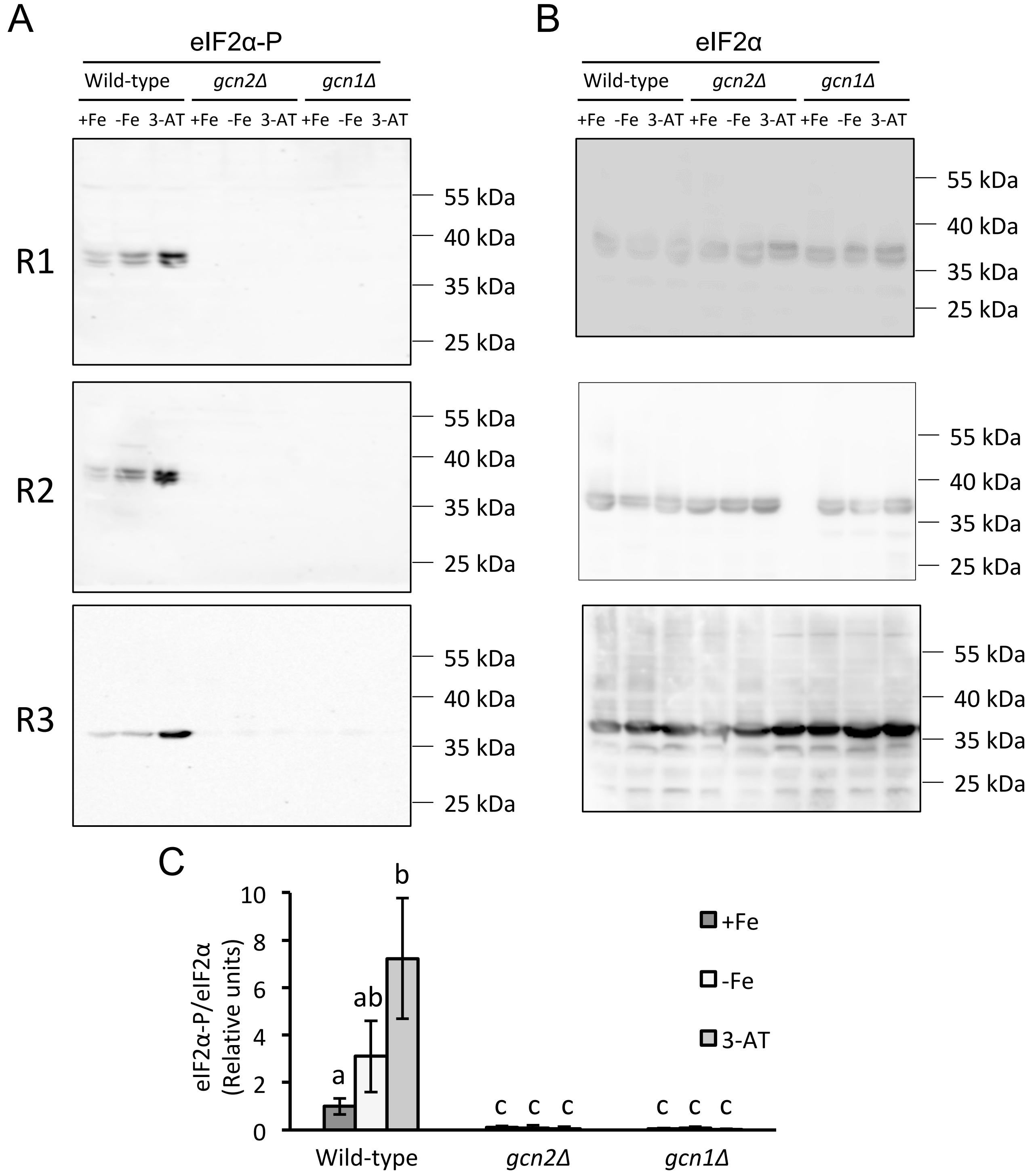

Supplementary Figure S3. eIF2α is not phosphorylated in the *gcn2*Δ and *gcn1*Δ mutant strains in response to iron deficiency or 3-aminotriazol treatment. Wild-type BY4741, *gcn2*Δ and *gcn1*Δ cells transformed with pRS413 plasmid were cultivated overnight and reioculated in SC-His (+Fe) or SC-His + 100 μM BPS (-Fe) for 9 hours, and in SC-His + 30 mM 3-AT for 5 hours. The levels of phosphorylated eIF2α (A) and total eIF2α (B) protein were determined by Western blot analyses in three independent biological replicates (R1, R2, R3) using the anti-eIF2α-Ser51/52 and anti-eIF2α antibodies, respectively. L: ladder. (C) Quantification of the relative levels of eIF2α-P/eIF2α. The average and standard deviation is shown. Different letters over the bars indicate statistically significant differences (p-value < 0.05).

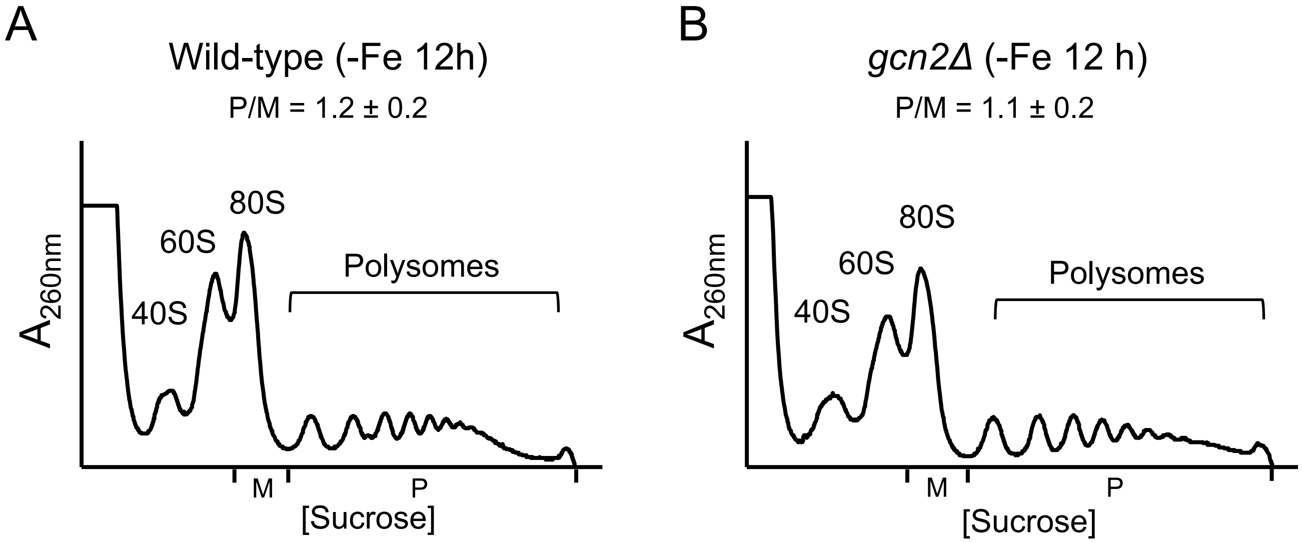

Supplementary Figure S4. The Gcn2-dependent improvement of translation under iron deficiency is temporary. Wild-type BY4741 **(A)** and *gcn2Δ* **(B)** strains were cultivated in SC with 100 µM BPS (-Fe) for 12 hours. Polysome analyses were performed as described in Figure 1.

Supplementary Table S1. List of *Saccharomyces cerevisiae* strains used in this work.

Strain	Description	Source
W303	HTLU-2832-1B: *MATa, HIS3, TRP1, LEU2, URA3, ADE2, can1*	Fred Cross
W303 ura3Δ	W303 *ura3::hphB*	This study
W303 ura3Δgcn2Δ	W303 *ura3::hphB gcn2::KanMX4*	This study
BY4741	*MATa, his3Δ1, leu2Δ0, met15Δ0, ura3Δ0*	Research Genetics
gcn2Δ	*BY4741 gcn2::KanMX4*	Research Genetics
gcn1Δ	*BY4741 gcn1::KanMX4*	Research Genetics
SUI2	RS-86: *MATa*, *ura3-52*, *leu2-3,112*, *trp1 Δ 63*, *Δ sui2*, *Δ p919 [SUI2, URA3]*, *pRS414 [SUI2, TRP1]*	32
SUI2-S51A	RS-88: *MATa*, *ura3-52*, *leu2-3,112*, *trp1Δ63*, *Δsui2*, *Δp919 [SUI2, URA3]*, *pRS414 [SU2I-S51A, TRP1]*	32

Supplementary Table S2. List of oligonucleotides used for RT-qPCR in this work.

Name	***Sequence (from 5' to 3')***
RPS16B-qPCR-F	GACGAACAATCCAAGAACGA
RPS16B-qPCR-R	AGAACGAGCACCCTTACCAC
RPL3-qPCR-F	CGAAGCTGTCACCGTTGTTG
RPL3-qPCR-R	AAATGTTCAGCCCAGACGGT
ACT1-qPCR-F	TCGTTCCAATTTACGCTGGTT
ACT1-qPCR-R	CGGCCAAATCGATTCTCAA
GCN4-qPCR-F	GACAACTTCATTCTTACCCACTCC
GCN4-qPCR-R	GATTCGTCATCCTTTCCAACA
